# Retroviral coinfection (Jaagsiekte and Maedi-Visna viruses) in sheep with pulmonary tumors in Transylvania (Romania): retrospective study on 82 cases

**DOI:** 10.3389/fvets.2024.1457971

**Published:** 2024-09-02

**Authors:** Dragoș Hodor, Corina Toma, Andrada Negoescu, Monika Olech, Dumitru Gordon, Cornel Cătoi, Marian Taulescu

**Affiliations:** ^1^Department of Anatomic Pathology, Faculty of Veterinary Medicine, University of Agricultural Sciences and Veterinary Medicine, Cluj-Napoca, Romania; ^2^Department of Pathology, National Veterinary Research Institute, Puławy, Poland; ^3^Department of Parasitology and Parasitic Diseases, Faculty of Veterinary Medicine, University of Agricultural Sciences and Veterinary Medicine of Cluj-Napoca, Cluj-Napoca, Romania

**Keywords:** ovine pulmonary adenocarcinoma, Maedi-Visna, coinfection, interstitial pneumonia, Romania

## Abstract

Ovine pulmonary adenocarcinoma (OPA) is an important viral-induced neoplasia in sheep caused by exogenous Jaagsiekte sheep retrovirus (exJSRV). Coinfection of exJSRV and Maedi-Visna virus (MVV) is reported in OPA cases, but its worldwide distribution and significance on lung pathology is not yet completely understood. This study aimed to investigate the MVV coinfection rate in 82 exJSRV-related OPA cases, and their pathological effects on lung parenchyma in slaughtered sheep in Transylvania (Romania). On gross examination, classical form of OPA was identified in 92.7%; no changes consisting with MVV interstitial pneumonia were identified in the included cases. The most common histological type of OPA was acinar (58.5%) and the myxoid growths were found in 18 cases. The exJSRV and MMV coinfection rate in examined sheep was 47.6% (39/82). The assessment of perineoplastic areas from coinfected animals, revealed interstitial lymphoplasmacytic infiltrates in all cases, lymphoid hyperplasia in 60.6% cases (20/33) and fibromuscular hyperplasia in 63.7% (21/33). This is the first report providing new data on distribution of OPA coexisting with MVV infection in slaughtered sheep in Romania. We consider that the OPA and MVV coinfection may play an important role on the severity of ovine chronic pulmonary diseases and further studies are needed to confirm this hypothesis.

## 1 Introduction

Retroviruses are a complex group of enveloped, double-stranded RNA viruses, which possess the unique ability to integrate their genetic material into the host genome, influencing numerous cellular functions and subsequently leading to a variety of pathologies (1).

In sheep flocks, retroviruses contribute to various diseases, including cancers such as exogenous Jaagsiekte Sheep Retrovirus (exJSRV)-related ovine pulmonary adenocarcinoma (OPA) and Enzootic Nasal Tumor Virus (ENTV)-related nasal tumors. In sheep and goats, chronic inflammatory respiratory diseases are caused by small ruminant lentiviruses (SRLVs) such as Maedi-Visna virus (MVV) and caprine arthritis encephalitis virus (CAEV) (2, 3).

OPA, also known as ovine pulmonary adenomatosis, is a chronic disease, characterized by neoplastic development of the lung parenchyma (4–6). JSRV is mainly transmitted by inhalation of viral particles, but the transuterine route and the ingestion of infected colostrum or milk were also reported. The gold-standard method for diagnosis of OPA is the post-mortem examination, followed by histological assessment of the affected areas, and JSRV is detected by immunohistochemistry (IHC) and/or polymerase chain reaction (PCR) (7). Macroscopically, OPA can be classified into classical and atypical forms, the latest being rarely encountered (8). Histologically, the adenocarcinomas are composed of both neoplastic type II pneumocytes and bronchial epithelial cells, associated with a moderate fibro-vascular stroma. The neoplastic stroma is often infiltrated by numerous lymphocytes, plasma cells, and macrophages; bronchus-associated lymphoid tissue (BALT) hyperplasia and alveolar histiocytosis are also often noticed (7).

Maedi-Visna (MV), also known as ovine progressive pneumonia (OPP), is an untreatable, chronic, viral disease mainly of sheep, with a worldwide distribution (9, 10).

The horizontal transmission route of MVV is caused by prolonged direct contact with infected animals or inhalation of infectious secretions, as well as via contaminated milking equipment. Vertical transmission is possible through the placenta or infected milk/colostrum. MVV produces a multisystemic syndrome affecting the lungs, joints, mammary gland, and central nervous system (CNS). Post-mortem examination of the lungs shows pale, considerably enlarged, and uniformly dense parenchyma, with rib imprints (11). Histologically, MVV infection of sheep lungs leads to lympho-plasmocytic interstitial pneumonia (LIP), lymphoid hyperplasia, interstitial fibrosis, and alveolar septal smooth muscle hypertrophy (12, 13). Effective diagnosis in the early stages of MV involves serological techniques such as, enzyme-linked immunosorbent assay (ELISA), agar gel immunodiffusion (AGID), Western Blot (WB), whereas PCR is used for effective detection of proviral DNA (9).

In small ruminants, the coinfection with both MVV and exJSRV have been previously investigated and described (4, 14–17), but the precise pathogenetic mechanisms underlying this coinfection are not fully understood. MVV and exJSRV coinfection may lead to synergistic or antagonistic interactions between the two viruses, potentially influencing pulmonary disease progression in the affected animals.

The objectives of this study were (1) to evaluate the coinfection rate of MVV in exJSRV-positive ovine pulmonary adenocarcinomas and (2) to describe the additional histological findings related to coinfection. This is the first report describing the occurrence of OPA coexisting with MVV in slaughtered sheep in Romania.

## 2 Materials and methods

The study was conducted over a period of 6 years (from 2017 to 2023) in an approved ovine slaughterhouse from the Transylvania region (central and northwestern Romania). All sheep included in this research were adult (between 2 to 4 years) females of the Ţurcană breed. The lungs were examined for specific gross features of OPA. Based on the macroscopic findings, the suspected OPA cases were classified into classical and atypical forms, according to García-Goti et al. and Toma et al. (8, 18).

Upon gross examination, lung tissue samples from 82 cases with features consistent with pulmonary tumors were documented and collected for further analyses. From each macroscopically suspected lung tumor, the samples were divided and either fixed in 10% buffered formalin for histological assessment or stored at −20°C for molecular analyses. In addition, lung samples (*n* = 10) with no gross lesions were collected as negative controls.

The selected tissue samples, including the tumor and peritumoral area, were stored in 10% buffered formalin for at least 48 h, routinely processed and embedded in paraffin. Sections of 2–3 μm thick were obtained and stained with hematoxylin-eosin (H&E). All samples were independently assessed using a light microscope by two veterinary pathologists (MT and DH) using a light Olympus BX-41 microscope and the photomicrographs were taken using an Olympus UC30 digital camera and Stream Basic imaging software (Olympus Corporation, Tokyo, Japan).

All lung tumors were histologically classified using the available criteria from the literature (19, 20). Histological findings compatible with SRLV-like lesions were also described according to previous studies (16). Negative control lungs (*n* = 10) without histopathological findings were also used.

DNA was extracted from 25 mg of each tissue sample using the Nucleospin Tissue kit (Macherey Nagel GmbH & Co KG, Duren, Germany) according to the manufacturer's instructions. The quality and quantity of DNA were assessed in a nanophotometer (Implen, Munich, Germany). For the detection of the proviral DNA of MVV, a nested real-time PCR was performed as previously described by Olech et al. (10). This newly developed PCR method can distinguish between CAEV and MVV, which are genetically closely related, and both belong to the SRLVs group. Therefore, all tested samples were tested with primers and probes designed for detection of the MVV-like and CAEV-like viruses. exJSRV proviral DNA was detected using the PCR method used by Toma et al. (18).

## 3 Results

All cases (*n* = 82), macroscopically suspected as lung tumors, were histologically confirmed as lung adenocarcinomas. The macroscopical, histological and molecular features of retroviral coinfection are detailed in Table 1.

**Table 1:** Macroscopic, histological and molecular features of exJSRV and MVV coinfection in slaughtered sheep in Romania.

**No. Crt**	**Sample code**	**Pathology**					**Molecular analysis**		
		**Macroscopic forms**	**Histological type**	**Additional findings in peritumoral area**			**exJSRV**	**MVV**	**CAEV**
				**Lymphoplasmacytic infiltrate**	**Lymphoid hyperplasia**	**Fibromuscular hyperplasia**			
1	33/21.12.17	Classical	Acinar	+	+	-	+	+	-
2	2/26.01.18	Atypical	Acinar	+	-	+	+	+	-
3	3/26.01.18	Classical	Acinar	+	-	+	+	+	-
4	12/26.01.18	Classical	Acinar	+	+	+	+	-	-
5	2/31.01.18	Classical	Papillary	N/A	N/A	N/A	+	-	-
6	5/31.01.18	Classical	Papillary	+	-	-	+	-	-
7	3/24.07.18	Classical	Papillary	N/A	N/A	N/A	+	+	-
8	5/24.07.18	Classical	Acinar + MGs	N/A	N/A	N/A	+	-	-
9	6/24.07.18	Classical	Papillary	N/A	N/A	N/A	+	-	-
10	7/24.07.18	Classical	Acinar	-	-	-	+	-	-
11	2/07.08.18	Classical	Papillary	+	+	+	+	+	-
12	3/07.08.18	Classical	Papillary + MGs	+	+	+	+	+	-
13	1/21.08.18	Classical	Acinar + MGs	+	+	+	+	+	-
14	2/21.08.18	Classical	Papillary	+	+	+	+	+	-
15	3/21.08.18	Classical	Acinar	+	+	+	+	+	-
16	5/21.08.18	Classical	Acinar	+	+	+	+	+	-
17	6/21.08.18	Atypical	MGs	+	-	-	+	+	-
18	1/12.10.18	Classical	Acinar	+	+	+	+	+	-
19	2/12.10.18	Classical	Papillary + MGs	+	-	-	+	-	-
20	3/12.10.18	Classical	Acinar	-	-	-	+	-	-
21	4/12.10.18	Classical	Acinar	+	-	-	+	+	-
22	5/12.10.18	Classical	Acinar	N/A	N/A	N/A	+	-	-
23	6/12.10.18	Classical	Acinar + MGs	+	+	+	+	+	-
24	7/12.10.18	Classical	Acinar	+	+	-	+	+	-
25	1/22.10.18	Classical	Acinar	-	-	-	+	-	-
26	3/07.05.19	Classical	Papillary + MGs	N/A	N/A	N/A	+	+	-
27	4/07.05.19	Classical	Acinar	+	-	-	+	-	-
28	1/22.10.21	Classical	Papillary	N/A	N/A	N/A	+	-	-
29	2/22.10.21	Classical	Papillary	+	+	-	+	+	-
30	4/22.10.21	Classical	Papillary	+	-	-	+	-	-
31	5/22.10.21	Atypical	Acinar	+	-	-	+	+	-
32	8/22.10.21	Classical	Papillary	+	-	-	+	+	-
33	10/22.10.21	Classical	Acinar	N/A	N/A	N/A	+	-	-
34	11/22.10.21	Classical	Papillary	N/A	N/A	N/A	+	-	-
35	12/22.10.21	Classical	Papillary + MGs	+	+	+	+	-	-
36	13/22.10.21	Classical	Papillary	N/A	N/A	N/A	+	-	-
37	14/22.10.21	Atypical	Acinar	+	-	-	+	-	-
38	15/22.10.21	Classical	Acinar	N/A	N/A	N/A	+	+	-
39	12MT+G	Classical	Acinar	+	+	+	+	-	-
40	22MT+G	Classical	Acinar	N/A	N/A	N/A	+	-	-
41	55MT+G	Classical	Acinar	+	-	+	+	+	-
42	70MT+G	Classical	Acinar	+	-	-	+	-	-
43	72MT+G	Classical	Acinar + MGs	+	+	-	+	-	-
44	78MT+G	Classical	Acinar	+	+	+	+	+	-
45	103MT+G	Classical	Acinar + MGs	+	-	+	+	+	-
46	110MT+G	Classical	Acinar	+	-	-	+	+	-
47	113MT+G	Classical	Acinar	+	-	-	+	-	-
48	114MT+G	Classical	Acinar	+	-	-	+	-	-
49	2/22.08.22	Classical	Acinar + MGs	+	+	+	+	-	-
50	3/22.08.22	Atypical	Acinar	+	-	-	+	-	-
51	5/22.08.22	Classical	Acinar	+	-	-	+	-	-
52	8/30.08.22	Classical	Acinar	+	+	-	+	+	-
53	11/30.08.22	Classical	Acinar	+	-	+	+	+	-
54	16/30.08.22	Classical	Acinar	+	+	+	+	+	-
55	19/30.08.22	Classical	Papillary	N/A	N/A	N/A	+	-	-
56	35/30.08.22	Classical	Acinar	N/A	N/A	N/A	+	-	-
57	1/05.10.22	Classical	Papillary	N/A	N/A	N/A	+	-	-
58	2/05.10.22	Classical	Acinar + MGs	+	+	+	+	+	-
59	3/05.10.22	Classical	Papillary	+	+	+	+	+	-
60	4/05.10.22	Classical	Acinar + MGs	N/A	N/A	N/A	+	-	-
61	5/05.10.22	Classical	Acinar	N/A	N/A	N/A	+	-	-
62	6/05.10.22	Classical	Acinar	N/A	N/A	N/A	+	+	-
63	7/05.10.22	Atypical	Acinar + MGs	+	-	-	+	-	-
64	8/05.10.22	Atypical	Acinar + MGs	+	-	-	+	-	-
65	9/05.10.22	Classical	Acinar + MGs	+	+	+	+	+	-
66	11/05.10.22	Classical	Acinar	+	-	-	+	+	-
67	12/05.10.22	Classical	Acinar + MGs	+	+	+	+	+	-
68	13/05.10.22	Classical	Acinar	+	-	-	+	-	-
69	14/05.10.22	Classical	Acinar	N/A	N/A	N/A	+	-	-
70	15/05.10.22	Classical	Acinar	+	-	+	+	+	-
71	16/05.10.22	Classical	Acinar	N/A	N/A	N/A	+	+	-
72	18/05.10.22	Atypical	Acinar	+	-	-	+	+	-
73	19/05.10.22	Classical	Acinar	N/A	N/A	N/A	+	+	-
74	20/05.10.22	Classical	Acinar	N/A	N/A	N/A	+	-	-
75	21/05.10.22	Classical	Acinar	+	-	-	+	-	-
76	22/05.10.22	Classical	Papillary	+	+	+	+	+	-
77	4/20.09.23	Classical	Acinar + MGs	-	-	-	+	-	-
78	6/20.09.23	Classical	Acinar	+	-	-	+	-	-
79	9/20.09.23	Classical	Acinar	-	-	-	+	-	-
80	12/20.09.23	Classical	Acinar	-	-	-	+	-	-
81	17/20.09.23	Classical	Acinar	+	+	-	+	+	-
82	18/20.09.23	Classical	Acinar	+	-	-	+	-	-

MGs, myxoid growth; N/A, not assessed.

**Figure 1:**
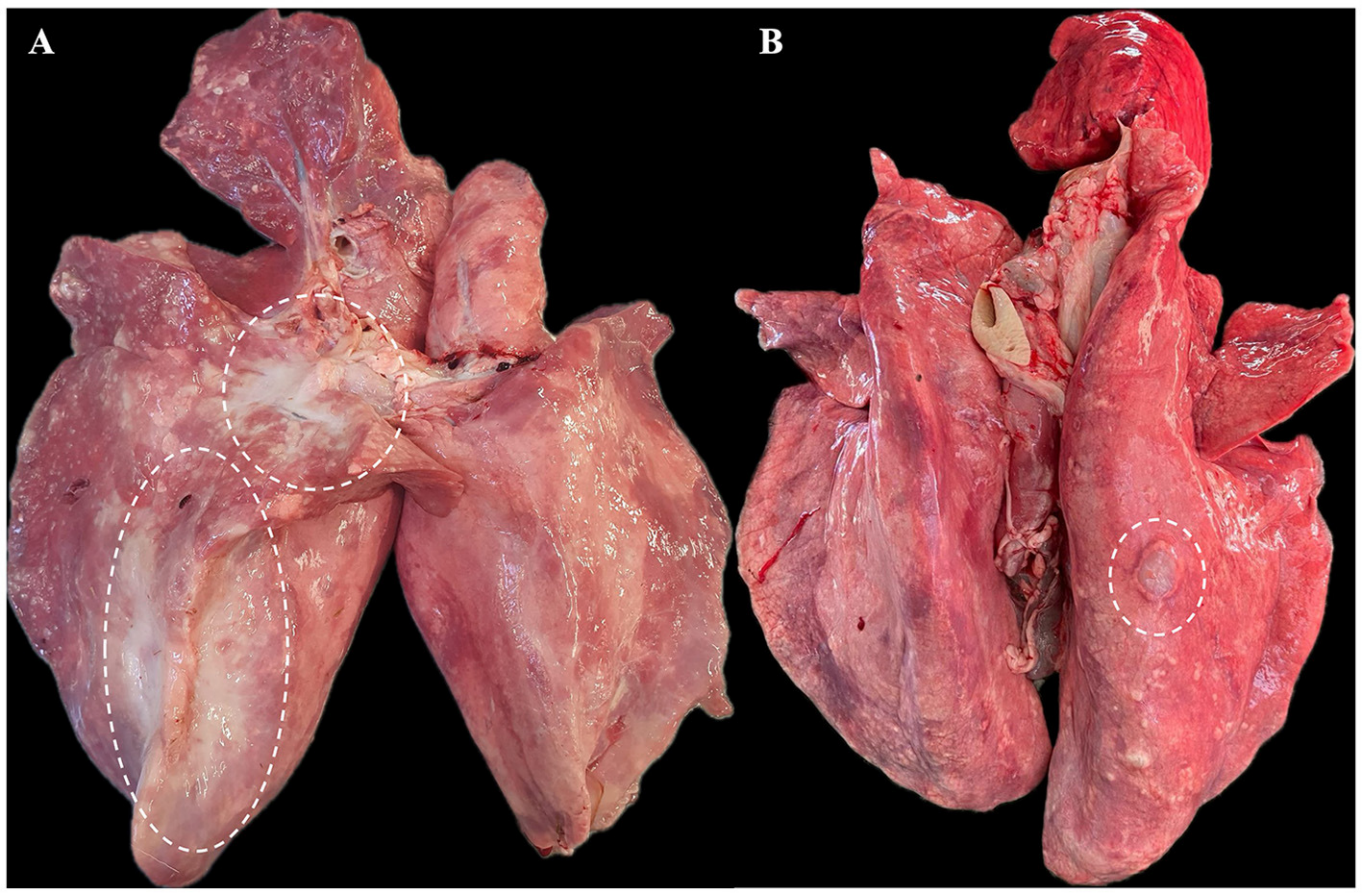
Gross features of pulmonary tumors. **(A)** Classical form of OPA - approximately 40% of the right lung parenchyma is replaced by a large, dense neoplastic mass (outlined with white dashes). **(B)** Atypical form of OPA - a single nodular mass of approximately 1.5–2 cm is present on the surface of the right diaphragmatic lobe.

Grossly, out of 82 cases, 74 lung tumors (74/82, 90.2%) were characterized by large irregular and poorly delimited masses, often affecting multiple lung lobes, predominantly involving the cranio-ventral areas of the lungs. The affected areas were dense on palpation, exhibiting white-grayish discoloration, and on cut-section, a moderate amount of foamy to viscous fluid was expressed. Thus, all these cases were classified as the classical form of OPA (Figure 1A). The remaining samples (8/82, 9.8%) were included in the atypical form of the disease (Figure 1B). These tumors were identified as single, well-delimited pearl-white dense masses, of 1–2 cm in diameter, located on the surface of the diaphragmatic lobes. Characteristic gross features of concurrent interstitial pneumonia were not observed in any of the evaluated lungs.

**Figure 2:**
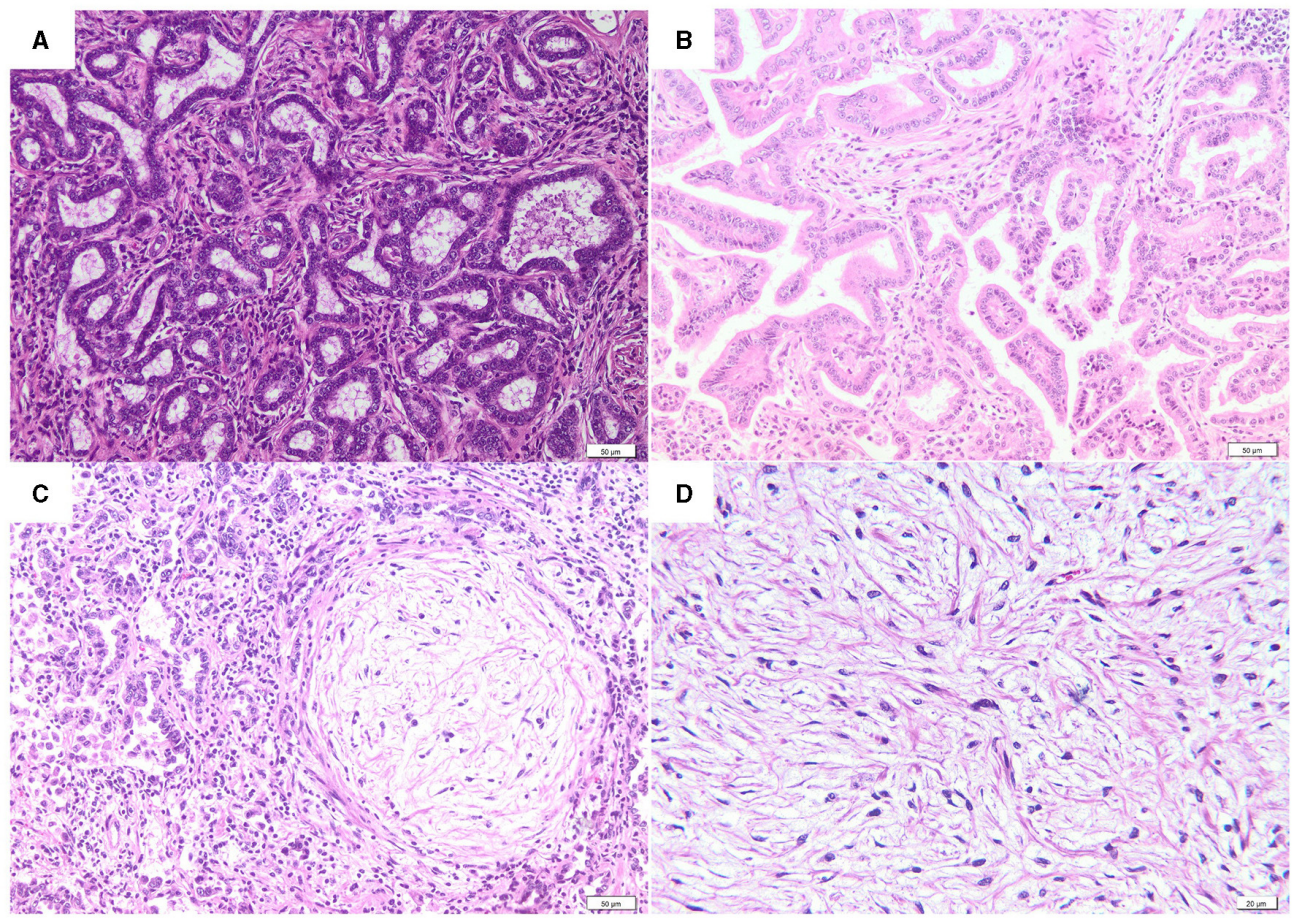
Histological features of OPA. **(A)** Acinar type (H&E, bar = 50μm). **(B)** Papillary type (H&E, bar = 50μm). **(C)** Acinar type with a myxoid growth nodule (H&E, bar = 50μm). **(D)** A detail of a myxoid growth nodule (H&E, bar = 20μm).

Histologically, most of the lung tumors (48/82, 58.5%), were classified as acinar type (Figure 2A). The acini were composed of well differentiated cubical to columnar neoplastic epithelial cells and supported by a moderate amount of fibrovascular stroma, often infiltrated with variable numbers of lymphocytes, plasma cells, and macrophages. A predominantly papillary type (Figure 2B) was observed in 16 (16/82, 19.5%) cases, characterized by the presence of tubular structures containing intraluminal papillary growths of cuboidal or columnar cells supported by a fibrovascular core. In thirteen cases of acinar type (13/82, 15.9%) and 4 cases of papillary type (4/82, 4.9%), the epithelial neoplastic component was multifocally to locally extensive intermingled with nodules of myxoid growths (MGs) (Figure 2C). The myxoid growths were sparsely cellular, composed of spindle-shaped neoplastic cells, organized in short streams, and separated by abundant extracellular matrix. Only one case (1/82, 1.22%) of pure myxoid growth (Figure 2D) without epithelial changes was diagnosed.

**Figure 3:**
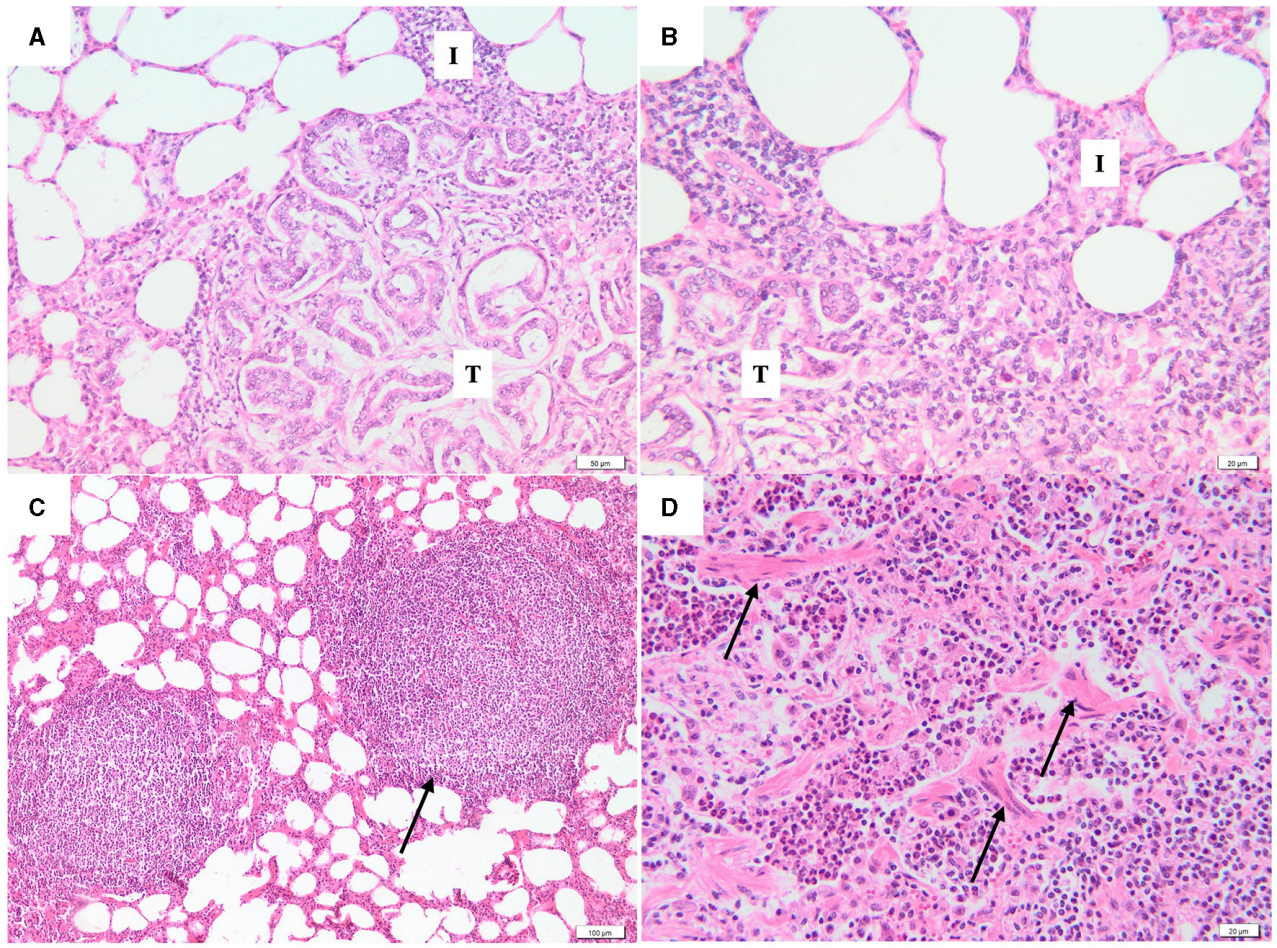
Photomicrographs of peritumoral area. **(A, B)** At the periphery of the neoplastic lesions (T), the alveolar septa are moderately thickened due to the presence of inflammatory infiltrates with lymphocytes and plasma cells (I) (H&E, bar = 50μm, 20μm). **(C)** Interstitial lymphoid hyperplasia (black arrow) without germinative center (H&E, bar = 100μm). **(D)** Fibromuscular proliferation (black arrows), characterized by irregular bundles of fibrous connective tissue and smooth muscle cells (H&E, bar = 20μm).

In addition to pulmonary neoplastic lesions, the peritumoral areas were evaluated for the presence of SRLV-like lesions, including interstitial lymphoplasmacytic infiltration, lymphoid hyperplasia, and fibromuscular hyperplasia. Interstitial lymphoplasmacytic infiltrate was observed in the peritumoral areas of 90% (54/60) of the samples (Figures 3A, B). Both lymphoid (Figure 3C) and fibromuscular hyperplasia (Figure 3D) were present in 41.7% (25/60) and 41.7% (25/60) of the studied cases, respectively. In 19 cases (31.7%) the lung showed both changes.

ExJSRV proviral DNA was detected by PCR in all included lung tumors. Of these, proviral DNA of MVV was detected in 39 samples (39/82, 47.6%), while CAEV proviral DNA was not detected in any of the evaluated samples.

Out of 39 coinfected pulmonary tissues, 6 cases (15.4%) were excluded due to lack of peritumoral area. In the remaining 33 cases, all cases showed interstitial inflammatory infiltrate. Lymphoid hyperplasia was identified in 20 cases and 21 lung tissues revealed fibromuscular hyperplasia. All the three lesions were identified in 15 cases.

## 4 Discussions

In veterinary medicine, retroviruses are associated with several diseases, including various forms of cancer, immunodeficiency syndromes and chronic inflammatory conditions. Retroviruses may cause a significant impact on sheep health and have important financial losses in sheep herds. In addition to the impact on animal health, these viruses and their associated diseases may represent good models for studying various related diseases in humans (21).

Ovine pulmonary adenocarcinoma and Maedi-Visna represent progressive wasting diseases with great economic importance in sheep industry (5, 22). In Romania, MV is a notifiable disease according to Annex 1 of Order of the President of the National Sanitary Veterinary and Food Safety Authority (NSVFSA) no. 79/2008, unlike OPA which has no regulations. The economic and welfare significance of these retroviral diseases of sheep has great importance as no treatment or effective disease prevention methods, such as vaccination, are available (9).

Currently, there is little information regarding the prevalence of these diseases in Romania. A few decades ago, the reported prevalence of OPA in Romania ranged from 0.5% to 0.8% (23, 24). However, a recent study, found that the prevalence of OPA has increased to 1.26% (18). Additionally, few studies investigated SRLV prevalence in Romania in both sheep and goats, but due to a relatively low number of animals included in these works, the epidemiological status of SRLV remains poorly known (25–29). However, a recent study published by Olech et al. marked the first description of SRLV strains circulating in Romania (10). Moreover, for the first time in Romania, our research highlighted the pulmonary lesions caused by coinfection of the two retroviruses.

A coinfection rate of 47.6% between the two retroviruses was reported in the present work. There are relatively few studies reporting coinfection between exJSRV and MVV, and the rates ranged from 6.19% to 11.1% (4, 16, 17). In addition, the present study analyzed a large number of cases, which may provide a better overview of coinfection rates. Nonetheless, the hypothesis of synergism between ovine lentiviruses and oncogenic retroviruses has been clarified by González et al. (15). The authors assumed that the synergistic effect mainly refers to an increased lateral transmission of MVV in coinfected animals, which is most probably explained due to the overproduction of lung fluid and the increased number of alveolar macrophages, both specific to OPA. The significance of the latter can be explained by the easier intrahistiocytic replication of SRLV (macrophages represents the target cells for SRLV) in OPA-positive cases (4, 30).

In a previous study, additional lesions of the peritumoral areas, known as SRLV-like lesions including presence of interstitial lymphocytic and plasmacytic infiltrate, formation of lymphoid nodules with germinal centers, interstitial fibrosis and hypertrophy of smooth muscle in the alveolar septa were identified in 25.8% (16), and these are in line with our findings.

Analysis of coinfection cases between both retroviruses showed a different inflammatory response associated with lymphoid and fibromuscular hyperplasia. These findings suggest that MVV infection may exacerbate the inflammatory process in the lungs, potentially contributing to the development of OPA. It has been reported that in human medicine, the development of lung cancer is associated with chronic inflammation which can stimulate various processes involved in tumor development such as cell proliferation, angiogenesis, and metastasis (31).

Nevertheless, some limitations of the study, such as the absence of data regarding the impact of retroviral coinfection on the clinical evolution in affected ewes, lack of testing for other pathogens with lung tropism, as well as lack of traceability of the origin of the cases, may limit the scientific quality of the present study and its translation into clinical practice.

ExJSRV and MVV coinfection may enhance the initial inflammatory response and its implications in the development of cancer. Clarifying this aspect may provide new insights into the oncogenic mechanism of OPA development and provide a good animal model for studying human lung cancer (32, 33).

## 5 Conclusions

The present study provides new data regarding the distribution of the exJSRV and MVV coinfection in sheep in Romania and worldwide, and this may play an important role in ovine chronic pulmonary diseases and lateral transmission of the viruses among individuals.

## Data Availability

The original contributions presented in the study are included in the article/supplementary material, further inquiries can be directed to the corresponding author.

## References

[1] Larruskain A Jugo BM. Retroviral infections in sheep and goats: Small ruminant lentiviruses and host interaction. Viruses. (2013) 5:2043–61. 10.3390/v5082043 23965529 PMC3761241

[2] Burmeister T. Oncogenic retroviruses in animals and humans. Rev Med Virol. (2001) 11:369–80. 10.1002/rmv.331 11746999

[3] Caswell JL Williams KJ. Respiratory system. In:Maxie G, editor. Jubb, Kennedy, and Palmer's Pathology of domestic animals, volume 2, New York: Elsevier Health Sciences (2016) 2016:477–8. 10.1016/B978-0-7020-5318-4.00011-5

[4] Ortega J Corpa JM Castillo D Murphy BG. Pathological spectrum of ovine pulmonary adenocarcinoma in small ruminants: a focus on the mixed form. Animals. (2023) 13:2828. 10.3390/ani13182828 37760228 PMC10525357

[5] De las Heras M Borobia M Ortín A. Neoplasia-associated wasting diseases with economic relevance in the sheep industry. Animals. (2021) 11:1–18. 10.3390/ani11020381 33546178 PMC7913119

[6] De las Heras M González L Sharp JM. Features of the clinical disease. In:Fan H, editor. Jaagsiekte Sheep Retrovirus and Lung Cancer. Berlin: Springer (2003).

[7] Quintas H Pires I Garcês A Prada J Silva F Alegria N. The diagnostic challenges of ovine pulmonary adenocarcinoma. Ruminants. (2021) 1:58–71. 10.3390/ruminants1010005

[8] García-Goti M Gonzá Lez L Cousens C Cortabarría N Extramiana AB Minguijó E. Characterization of two pathological forms associated with jaagsiekte retrovirus. J Compar Pathol. (2000) 122:55–65. 10.1053/jcpa.1999.0344 10627391

[9] Kalogianni AI Bossis I Ekateriniadou LV Gelasakis AI. Etiology, epizootiology and control of maedi-visna in dairy sheep: a review. Animals. (2020) 10:616. 10.3390/ani10040616 32260101 PMC7222820

[10] Olech M Hodor D Toma C Negoescu A Taulescu M. First molecular characterization of small ruminant lentiviruses detected in Romania. Animals. (2023) 13:3718. 10.3390/ani13233718 38067069 PMC10705781

[11] López A Martison SA. Respiratory system, mediastinum, and pleurae. In:Zachary JF., editor *Pathologic Basis of Veterinary Disease 7th edition*. New York: Elsevier Health Sciences (2021). p. 620–621.

[12] Gayo E Polledo L Balseiro A Martínez CP García Iglesias MJ Preziuso S. Inflammatory lesion patterns in target organs of visna/maedi in sheep and their significance in the pathogenesis and diagnosis of the infection. J Comp Pathol. (2018) 159:49–56. 10.1016/j.jcpa.2018.01.001 29599005

[13] Minguijón E Reina R Pérez M Polledo L Villoria M Ramírez H. Small ruminant lentivirus infections and diseases. Vet Microbiol. (2015) 181:75–89. 10.1016/j.vetmic.2015.08.007 26371852

[14] Dawson M Done SH Venables C Jenkins CE. Maedi-visna and sheep pulmonary adenomatosis: a study of concurrent infection. Br Vet J. (1990) 146:531–8. 10.1016/0007-1935(90)90056-9 2176908

[15] González L Juste RA Cuervo LA Idigoras I De Ocariz CS. Pathological and epidemiological aspects of the coexistence of Maedi-Visna and sheep pulmonary adenomatosis. Res Vet Sci. (1993) 54:140–146. 10.1016/0034-5288(93)90049-L 8384727

[16] Rosato G Abril C Hilbe M Seehusen F. A combined approach for detection of ovine small ruminant retrovirus co-infections. Viruses. (2023) 15:376. 10.3390/v15020376 36851589 PMC9958757

[17] Valecha S Roopa N Yadav HS Singh R Singh V Kumar P. Co-infection of maedi visna virus (MVV) with jaagsiekte sheep retrovirus (JSRV) and mycoplasma in Indian sheep and Goats. Indian J Vet Pathol. (2023) 47:13–17. 10.5958/0973-970X.2023.00002.0

[18] Toma C Bâlteanu VA Tripon S Trifa A Rema A Amorim I. Exogenous Jaagsiekte Sheep Retrovirus type 2 (exJSRV2) related to ovine pulmonary adenocarcinoma (OPA) in Romania: prevalence, anatomical forms, pathological description, immunophenotyping and virus identification. BMC Vet Res. (2020) 16:1–15. 10.1186/s12917-020-02521-1 32807166 PMC7433209

[19] Wilson DG. Tumors of the respiratory tract. In:Meuten DJ., Editor *Tumors in Domestic Animals*. Ames, IA, USA: John Wiley & Sons Inc. (2017). p. 467–498. 10.1002/9781119181200.ch12

[20] De las Heras M De Martino A Borobia M Ortín A Álvarez R Borderías L. Solitary tumours associated with jaagsiekte retrovirus in sheep are heterogeneous and contain cells expressing markers identifying progenitor cells in lung repair. J Comp Pathol. (2014) 150:138–147. 10.1016/j.jcpa.2013.09.001 24176105

[21] Leroux C Mornex JF. Retroviral infections in sheep and the associated diseases. Small Rumin Res. (2008) 76:68–76. 10.1016/j.smallrumres.2007.12.010

[22] de Miguel R Arrieta M Rodríguez-largo A Echeverría I Resendiz R Pérez E. Worldwide prevalence of small ruminant lentiviruses in sheep: A systematic review and meta-analysis. Animals. (2021) 11:1–21. 10.3390/ani11030784 33799908 PMC8000744

[23] Adame steanu C Baba AI Vesa S Rotaru O Mican V. Pulmonary adenomatosis of sheep (in Roumania). Lucrări tiintifice Institutul Agronomic Dr Petru Groza Cluj, Seria Medicină Veterinară. (1970) 26:87–92.32807166

[24] Baba AI Rotaru O Gaboreanu M Sissoko I. Post-mortem findings in pulmonary adenomatosis of sheep in Romania. In: Lucrările sectiei de patologie la taurine , si ovine. (1980). p. 357–362.

[25] Gurău MR Baraitareanu S Daneş D. Serological survey of caprine arthritis-encephalitis virus infection in a Southeastern Romanian farm. Sci Works Ser C Vet Med. (2015) 61:169–71.26498401

[26] Enache DA Baraitareanu S Dan M Gurau MR Otelea F Dobre A. Preliminary results of MVV and CAEV seroprevalence in Romanian sheep and goats. Sci Works C, Vet Med. (2017) 63:6.

[27] Mihai I Crivei IC Horhogea C Savu t a G Velescu E. Preliminary serological investigation on caprine arthritis and encephalitis virus infection in a goat farm from north-eastern Romanian region. Bull Univ Agric Sci Vet Med Cluj-Napoca. (2018) 75:243. 10.15835/buasvmcn-vm:2017.0061

[28] Mihai I Velescu E Tanase OI. Epidemiological observations on infectious pathology of goats in the Northeast area of Romania. In: Agriculture for Life, Life for Agriculture, Conference Proceedings. (2018). p. 449–454. 10.2478/alife-2018-0069

[29] Potârniche AV Cerbu C Olah D Suatean M Peredi C Guranda S. Serological survey of caprine arthritis-encephalitis virus infection in Sibiu county, Romania. Sci Works Ser C Vet Med. (2018) 64:70–2.26498401

[30] Griffiths DJ Martineau HM Cousens C. Pathology and pathogenesis of ovine pulmonary adenocarcinoma. J Comp Pathol. (2010) 142:260–83. 10.1016/j.jcpa.2009.12.013 20163805

[31] Budisan L Zanoaga O Braicu C Pirlog R Covaliu B Esanu V. Links between infections, lung cancer, and the immune system. Int J Mol Sci. (2021) 22:9394. 10.3390/ijms22179394 34502312 PMC8431665

[32] Gray ME Meehan J Sullivan P Marland JRK Greenhalgh SN Gregson R. Ovine pulmonary adenocarcinoma: a unique model to improve lung cancer research. Front Oncol. (2019) 9:335. 10.3389/fonc.2019.00335 31106157 PMC6498990

[33] Youssef G Wallace WAH Dagleish MP Cousens C Griffiths DJ. Ovine pulmonary adenocarcinoma: a large animal model for human lung cancer. ILAR J. (2015) 56:99–115. 10.1093/ilar/ilv014 25991702

